# A first remotely-delivered guided brief intervention to reduce intrusive memories of psychological trauma for healthcare staff working during the ongoing COVID-19 pandemic: Study protocol for a randomised controlled trial

**DOI:** 10.1016/j.conctc.2022.100884

**Published:** 2022-01-12

**Authors:** Laura Singh, Marie Kanstrup, Beau Gamble, Anahita Geranmayeh, Katarina E. Göransson, Ann Rudman, Oili Dahl, Veronica Lindström, Anna Hörberg, Emily A. Holmes, Michelle L. Moulds

**Affiliations:** a Department of Psychology, Uppsala University, Uppsala, Sweden; b Swedish Collegium for Advanced Study, Uppsala, Sweden; c Division of Psychology, Department of Clinical Neuroscience, Karolinska Institutet, Stockholm, Sweden; d Medical Unit for Medical Psychology, Karolinska University Hospital, Stockholm, Sweden; e Emergency and Reparative Medicine Theme, Karolinska University Hospital, Stockholm, Sweden; f Department of Medicine, Solna, Karolinska Institutet, Stockholm, Sweden; g School of Health and Welfare, Dalarna University, Falun, Sweden; h Division of Nursing, Department of Neurobiology, Care Sciences and Society, Karolinska Institutet, Huddinge, Sweden; i Department for Health Promoting Science Sophiahemmet University Stockholm, Sweden; j Samariten, Ambulance Stockholm, Sweden; k School of Psychology, The University of New South Wales, UNSW Sydney, Australia

**Keywords:** COVID-19, Digital intervention, Healthcare staff, Intrusive memories, Psychological trauma, Randomised controlled trial

## Abstract

Addressing the mental health needs of healthcare staff exposed to psychologically traumatic events at work during the COVID-19 pandemic is a pressing global priority. We need to swiftly develop interventions to target the psychological consequences (e.g., persistent intrusive memories of trauma). Interventions for healthcare staff must be brief, flexible, fitted around the reality and demands of working life under the pandemic, and repeatable during ongoing/further trauma exposure. Intervention delivery during the pandemic should be remote to mitigate risk of infection; e.g., here using a blend of digitalized self-administered materials (e.g., video instructions) and guided (remote) support from a researcher. This parallel groups, two-arm, randomised controlled trial (RCT) with healthcare staff working during the COVID-19 pandemic is the first evaluation of whether a digitalized form of a brief cognitive task intervention, which is remotely-delivered (guided), reduces intrusive memories. Healthcare staff who experience intrusive memories of work-related traumatic event(s) during the COVID-19 pandemic (≥2 in the week before inclusion) will be randomly allocated (1:1) to receive either the cognitive task intervention or an active (attention placebo) control, and followed up at 1-week, 1-month, 3-months, and 6-months post-intervention. The primary outcome will be the number of intrusive memories reported during Week 5; secondary and other outcomes include the number of intrusive memories reported during Week 1, and other intrusive symptoms. Findings will inform further development and dissemination of a brief cognitive task intervention to target intrusive memories.

## 1 Introduction

The COVID-19 pandemic highlights the global need for scalable and remotely delivered psychological interventions to protect the mental health of healthcare staff exposed to psychological trauma at work [1]. Repeated trauma exposure may have serious psychological consequences including symptoms related to posttraumatic stress. Brief interventions targeting such symptoms are needed.

Intrusive memories are common following traumatic events: they are repetitive, come to mind involuntarily, comprise primarily visual mental images, and typically contain ‘worst moment’ scenes (‘hotspots’) of the trauma. Intrusive memories can be distressing in their own right [2] and impair functioning (e.g., concentration [3]). They comprise a core feature of posttraumatic stress disorder (PTSD) [4]. Before the pandemic, intrusive memories of work-related traumatic events have been reported in a high proportion of emergency nurses [5]. During the pandemic, high and increased levels of symptoms related to PTSD have been documented in healthcare workers globally [[6], [7], [8], [9]], e.g. 40% of ICU staff [9]. PTSD symptoms have been linked to impaired work functioning [10], job termination [11], and burnout [12]. Whilst empirically-supported treatments for PTSD exist, we lack evidence-based interventions suitable for individuals experiencing ongoing/recurrent trauma (such as healthcare staff), as well as interventions targeting specific symptoms [13]. Intrusive memories provide a target for early intervention [2,14].

Our early laboratory studies informed the development of a brief cognitive task intervention to reduce intrusive memories after trauma [16]. It is an imagery-competing task intervention, designed to target intrusive memories on the basis that they predominantly take the form of visual mental images (e.g., the sight of a tube by a hospital bed). The task includes a brief memory reminder cue followed by Tetris gameplay (at least 20 min, with instructions to employ ‘mental rotation’), alongside monitoring the target symptom. As we have outlined elsewhere [17], in the intervention the content of the specific intrusive memory must be made active in memory using a retrieval cue (e.g., by selecting one image at a time from an individual ‘hotspot’ list of intrusive memories and briefly visualizing/‘seeing’ it in the mind's eye). Following activation, we predict that engaging in a visuospatial task during a critical time window will compete for working memory resources with the visual mental imagery component of the specific intrusive memory, and thus interfere with and limit its re-storage, and in turn reduce the occurrence of intrusive memories of the specific ‘hotspot’.

This prediction is consistent with the findings of dual-task experiments, which indicate that when similar cognitive tasks compete for shared resources, they interfere with one another and thereby impede memory processing; e.g., a visuospatial pattern-tapping task interfered with holding a visual mental image in mind, rendering it less vivid and emotional, whereas counting numbers aloud did not [18]. Such a dual-task capacity limitation provides an advantage by which to limit resources allocated to a maladaptive intrusive, image-based memory. Accordingly, a visuospatial task (such as playing the computer game Tetris using mental rotation) performed when the specific memory is in mind (via selection from the list of intrusive memories) should interfere with visual memory restorage (when the memory is modifiable), and thereby reduce the number of subsequent intrusions.

Relative to control, an earlier form of our brief cognitive task intervention (delivered by a researcher in person) prevented intrusive memories when delivered soon following traumatic birth [19] and motor vehicle accidents [20]. Adapted for older trauma, the intervention reduced the number of intrusions in case series including in-patients with complex PTSD [21] and refugees [22], and an individual with bipolar disorder [23].

We recently conducted feasibility [24] and pilot [25] studies of this brief cognitive task intervention with patients admitted to the Emergency Department (ED) in Sweden. The intervention resulted in 48% fewer intrusions relative to control at Week 1, and 90% fewer at Week 5 [25]. After commencing an RCT with ED patients (terminated due to the COVID-19 pandemic [26]) and on hearing healthcare staff's calls for interventions, we swiftly modified study procedures for remote delivery to *healthcare staff* working during COVID-19 [27], acknowledging their urgent need [1].

Given the demanding working lives of healthcare staff, interventions may have most benefit if they are brief, flexible, simple, and allow subsequent self-administration (i.e., are repeatable during ongoing/further trauma exposure). During the pandemic healthcare staff were working more (and longer) shifts than usual, and thus had less flexibility and less time for appointments. They faced multiple additional challenges, including the sheer burden of work, the need to wear personal protective equipment (PPE), a lack of breaks, and difficulty finding a private space to talk or to access resources such as desktop computers. Accordingly, we adapted this intervention to be brief (one main guided session), require minimal contact with researchers, and for delivery on the user's own smartphone at a time and place suitable to each participant. We created flexibility in the timing of phone calls with researchers (including evenings/weekends), and in how data were collected, and participants did not need to book multiple sessions or travel to attend appointments. These adaptations were made after consultation with healthcare staff collaborators, and also informed by small scale pilot work [15]. Specifically, we piloted a remotely-delivered and guided blended digitalized form of the intervention with three nurses experiencing intrusive memories during COVID-19 [15]. All three reported zero intrusions during Week 5; study procedures were feasible and acceptable.

The current trial with healthcare staff who experienced work-related traumatic events during the ongoing pandemic will evaluate whether a form of the brief cognitive task intervention reduces the number of intrusive memories compared to attention placebo control. For the first time, both will be delivered in a digital (blend of digitalized materials) and remotely-delivered form guided by researchers, with the aim of fitting study procedures around the challenges of healthcare staff's working lives under the pandemic. Both groups will complete daily digital intrusion diaries (symptom monitoring) at Week 0, Week1 and Week 5. Primary outcome is the number of intrusive memories during Week 5 post-intervention, with follow-ups at 1-week, and 1-, 3- and 6-months.

## 2 Materials and methods

### 2.1 Design

This intervention study is a parallel-groups, two-arm randomised controlled trial (1:1 allocation ratio) comparing a brief cognitive task intervention in its first digital (using a blend of digitalized materials) and remotely-delivered (guided) form with an attention placebo control in reducing the number of intrusive memories during Week 5 post-intervention. After a telephone assessment to determine eligibility for inclusion in the study (before randomisation), all participants will complete a baseline diary daily for seven days (intrusive memories diary Week 0). On Day 1, participants will complete baseline questionnaires (including demographic information and trauma history) before randomisation. The primary outcome (number of intrusive memories) will be assessed during Week 5 post-intervention. See Table 1 for an overview of the schedule of assessments for secondary and other outcomes. A measure of adverse events will be completed at all follow-ups.

Owing to the COVID-19 pandemic and risk of infection, all assessments/data collection will take place using remote/digital means rather than in-person. Data collection methods will be adapted to suit participant preference (e.g., format: self-assessment forms in an electronic format - via smartphone or computer - or paper sent via post; frequency: the frequency of prompts to assess the number of intrusive memories per week can be adapted to participant preference, between four per day to one per week). Possible methods for the digital meetings with research personnel include telephone or video call. Baseline assessment and guided delivery of the single session intervention (or control task) will be conducted remotely using a blend of digital tools and guided (remote) support from a researcher. After the guided session the intervention can be self-administered and participants will be encouraged to use it independently. Optional self-administered booster sessions can be conducted by the participant (self-guided), with the option of remote researcher support (researcher-guided).

#### 2.1.1 Hypotheses

Our primary hypothesis is that participants in the intervention arm, relative to those in the (attention placebo) control arm, will report fewer intrusive memories in the diary during Week 5 post-intervention. Our secondary hypotheses include that participants in the intervention arm, relative to those in the control arm, will report fewer intrusive memories during Week 1 and lower levels of intrusive symptoms (measured by the IES-R intrusion subscale and Posttraumatic Stress Disorder Checklist 5).

### 2.2 Eligibility criteria

To be eligible to take part in the study, participants must: be aged 18 or over, have carried out clinical work during the COVID-19 pandemic in hospital and care facilities (e.g., ICU, ambulance, intermediate care, ward) and experienced at least one traumatic event in relation to their clinical work during the pandemic. The traumatic event/s must satisfy the DSM-5-PTSD Criterion A definition of trauma (i.e., exposure to actual or threatened death, serious injury, or sexual violence by “Directly experiencing the traumatic event(s)" or “Witnessing, in person, the event(s) as it occurred to others”), and must have occurred since the start of the COVID-19 pandemic. Participants must also report distressing intrusive memories of this event, and have experienced at least two such intrusive memories in the previous week. Participants must also be able and willing to briefly write down these memories (without going into detail).

In line with our previous research with patients in the ED, participants must also report a full memory of the traumatic event, be alert and orientated, have access to an internet enabled smartphone and sufficient mobility to use it, be fluent in spoken and written Swedish, be willing and able to provide their informed consent and complete study procedures, and be willing and able to be contacted whilst the study is ongoing. Any individuals who lost consciousness for >5 min in relation to the traumatic event or who were intoxicated during the traumatic event will be excluded.

Receiving other care or interventions during the trial is allowed during participation.

### 2.3 Recruitment

The study will be advertised via posters and information materials posted in health care facilities in Sweden, digital hospital resources, social media, a study webpage, newspapers and other channels suggested by healthcare staff. Local networks at different healthcare facilities in Sweden have agreed to support recruitment by advertising the study to staff. Posters and flyers will be placed on hospital noticeboards and will include links to a study webpage with study information. A potential barrier for recruiting the sample for this RCT could be the heavy burden placed on healthcare staff during the pandemic (e.g., working extremely long/additional shifts, overtime, few or no days off, fatigue), which could prevent them from engaging in a research study. Thus, recruitment materials will be refined and optimized, if necessary (e.g., design of study website; in addition to digital materials, using paper recruitment posters when pandemic conditions allow).

Potential participants will be able to sign-up to take part by contacting the research group via a study-specific email address. The researchers will then contact prospective participants by telephone in order to answer any questions, provide further information about the study as necessary, conduct the eligibility assessment, and obtain digital written (scanned paper) or verbal (audio-recorded during phone call) and informed consent (see Appendix 1 for participant information and informed consent sheet). For more detail about the informed consent procedures, see section 5, Ethical Considerations*.*

### 2.4 Randomisation and concealment

Randomisation will take place on Day 1 after participants have completed baseline assessments and immediately before the intervention/control session is scheduled. Participants will be randomised to either the intervention or control arm by a randomisation tool (from SMART-TRIAL, the electronic platform used for study procedures/data collection), which uses permuted block randomisation with random blocks of the size 2–10. The pseudorandom number generator is from https://www.npmjs.com/package/seedrandom (see help. smart-trial.co for further details). Randomisation will be computerised and automated to ensure allocation concealment, and participants will be enrolled into the assigned condition by the researchers in the electronic platform.

Participants will not be informed as to whether they have been allocated to the intervention or control condition, or about the nature of the two conditions. That is, the participant information statement will only explain that two different tasks will be compared in the study, without revealing that one is an attention placebo task. In addition, participants will only be given details of the condition to which they are allocated. Owing to the nature of the intervention, the researcher administering the intervention cannot be blind to condition. The data analysts will be blind to participant condition (i.e., groups will be labelled only as ‘A’ and ‘B’ in the analysis data file) and any blinding breaks will be reported.

### 2.5 Intervention/control procedures

#### 2.5.1 Both conditions: information and baseline symptom monitoring in a diary (Week 0)

Via remote researcher guidance as well as two brief animated videos (accessed through links leading to an external website where the videos are stored), participants in both conditions will receive information about the target symptom (intrusive memories, animated video, 2:25 min) and information about how to monitor and report this symptom (animated video, 1:50 min). They will be asked to complete a digitalised intrusive memory diary in the electronic platform SMART-TRIAL [28]; i.e., monitor the occurrence of any intrusive memories during Week 0 (see 2.6.3).

#### 2.5.2 Both conditions: baseline assessment, randomisation and digitalized study materials/instruction videos (Day 1)

After completing the diary and baseline assessments (data collected remotely/digitally, e.g., computer or smartphone, via a secure platform [28]), participants will be randomised to the intervention or control task. Both tasks will be administered remotely; i.e., in a blended digitalized form with remote researcher support. That is, for the first time, study procedures will be delivered in a digitalized form and participants will be required to navigate between various guided online components. These include instructions and assessments presented in the electronic data collection platform SMART-TRIAL, links to instructional videos (both animated videos [27] and recorded videos of a researcher providing verbal instructions), and external websites to access the intervention/control task on the participant's phone (see section 2, 2). Both groups will answer brief quiz questions in the electronic platform SMART-TRIAL after they have watched the videos (e.g., about what they are instructed to do during the task), in order to reinforce their understanding of how to complete the study procedures. The researcher aims to be present on phone/video call for the full duration of the initial dose of the intervention/control task session to promote adherence, clarify and answer questions that may arise and guide participants through the session, and provide guidance as necessary regarding how to navigate the various digital interfaces (secure electronic platform, videos accessed via links, websites etc.).

#### 2.5.3 Intervention condition: first dose of the intervention is guided with researcher support (Day 1)

The first intervention session will be guided remotely by a trained researcher and includes the following three components: (1) A **brief memory reminder cue**; (2) engaging in a **visuospatial interference task for at least 20 min**; and (3) actively **using ‘mental rotation’** during the gameplay.

First, participants will be given information about the intervention and how it should be carried out (video of a researcher, 1:51 min, researcher available for questions/clarifications). They will be informed that the target image of the traumatic event needs to be active in memory before gameplay, and that they need to play the game in a particular way (i.e., using mental rotation).

*List of intrusive memories – memory reminder cue procedure.* Then, participants will watch a video of a researcher (2:03 min) explaining how to complete a list of their intrusive memories which involves briefly describing ‘what they see’ when they experience each intrusive memory. This element will be guided by a researcher who will also ask the participant to write a brief description of each memory (e.g., ‘face of a patient who cannot breathe’) into the electronic data collection platform, one image at a time until each intrusive memory has been described. At no stage will participants be asked to describe the trauma in detail. Participants will then be asked to choose one intrusive memory from their list and gently bring that target image to mind; i.e., focus briefly on one intrusive image so that it becomes active in working memory as a visual mental image.

*Tetris gameplay with mental rotation and recap.* After this memory reminder cue procedure, participants will watch an animated video explaining how to play Tetris using mental rotation (2:43 min, accessed via a link in the electronic platform leading to an external website where the video is stored). They will then access the game Tetris® (either by clicking on a link in the electronic platform or by inserting www.tetris.com in the web browser of their smartphone), skipping any advertisement that comes up in this free version of Tetris, and adjusting the settings to ‘ghost piece off' (c. 2–3 min). Participants will then be encouraged to play the game for at least 20 min, whilst actively using ‘mental rotation’ throughout the gameplay, i.e., planning ahead and visualizing in their mind's eye how to rotate and move upcoming Tetris blocks to fit them into a horizontal line. During gameplay, the researcher will provide support as needed and monitor the time spent playing.

After gameplay, participants will watch a final animated video (1:37 min, accessed via a link in the electronic platform leading to an external website where the video is stored) which re-caps the procedure and includes an illustration of what is happening in the brain [29] during the task. This information is included to provide participants with a re-cap on what they have done during the session and - in keeping with the approach of established psychological interventions (e.g., cognitive behaviour therapy) - to provide participants with a rationale for the intervention, and some brief information about the hypothesized mechanisms. Further, the video re-emphasizes that it is important to use mental rotation when playing Tetris, as mental rotation can be one of the more difficult aspects of the gameplay instructions to grasp. That is, participants are reminded that they should imagine the upcoming blocks in their mind's eye (rather than just focus on the single block entering the game and simply swiping as they might usually do). The video highlights where in the brain the process of mental rotation happens. Overall, this last video aims to help participants remember the rationale for and components of the intervention that they can use later in a self-administered booster.

*Explanation of booster sessions for persistent intrusions or for different intrusions on the list.* After the first ‘dose’, the intervention can be repeated independently for the remaining intrusive memories on the participant's list, and/or if the targeted memory were to persist. Thus, in this last phase of the guided session, the researcher will explain how to use the intervention in a self-administered way; e.g., encourage participants to use it after they experience an intrusive memory in daily life (i.e., when an image comes to mind involuntarily) if it is convenient to do so. It may be the case that in many instances, it is not feasible for participants to use the intervention immediately after experiencing an intrusion (e.g., if they are at work). Accordingly, another option for participants who experience persistent intrusions would be for them to find a more convenient time when they can *deliberately* bring the intrusive image to mind (via the memory reminder cue procedure). Whether the memory is involuntarily or deliberately retrieved, participants will be encouraged to next play Tetris with active mental rotation for at least 20 min continuously. They are not expected to do the task procedure each and every time they have an intrusion, rather it is participants' choice as to how they can flexibly fit boosters into their daily life. We also note that the intervention is to be conducted once for each different intrusive memory (i.e., from participants' *list of intrusive memories*) – thus if the same memory intruded 10 times, the booster would be done once. However, if two different memories intruded, two boosters would be needed. If a given memory continues to persist, then a booster could be done again. Participants can also receive researcher-guided ‘Booster doses’ (i.e., with remote researcher support, see 2.6.5).

*Duration of the intervention.* The guided intervention task procedure conducted with researcher support will be approximately 25 min in duration; when self-administered the intervention takes c. 20 min.

#### 2.5.4 Control condition: first dose of control task is guided with researcher support (Day 1)

As per the intervention condition, the first session will be guided remotely by a trained researcher. Participants in the control condition will be instructed to listen to a podcast with neutral content about theoretical aspects of philosophy (the episode ‘Vägen till en svensk filosofi’ of the podcast ‘Filosofiska Rummet’ [30], accessed either by clicking on a link in the electronic platform or by inserting www.sverigesradio.se/avsnitt/1073596 in the web browser of their smartphone) for at least 20 min. They will watch a video of a researcher describing the task (0:53 min), i.e., to listen uninterrupted to the podcast for at least 20 min and focus on what is being said. Participants will be told that they can continue listen to the episode on their own later on. They will then receive information about how to access the podcast audio file. The researcher will provide support as needed and will monitor the time while the participant is listening to the podcast. This comparator was chosen based on pilot work [25] and is intended to match the intervention condition for attention demands, expectation effects, delivery device (smartphone), time spent with the researcher, as well as the need to navigate the blended digital materials (electronic platform and links to external websites) in this remote setup.

#### 2.5.5 Booster procedures

Participants in both conditions will be informed that they can contact the research team at any time during the study; thus, will have the option to e.g., ask the research team for researcher-guidance, including advice about the repeated use of the task procedure, or to ask any other questions they may have about the study procedures. In the first researcher-guided session, participants in both conditions will be informed that they can repeat the task procedure on their own (i.e., option for self-administered booster doses).

Participants in both conditions will be asked to monitor the number of intrusions they experience in the week following the first session (i.e., Week 1 diary) and can receive reminders from the research team if they stop filling in the diary (see 2.6.6).

As described above (see 2.6.3), participants in the intervention condition will be encouraged to repeat the intervention if they experience persistent intrusive memories. They could do this in two ways – either (a) use the gameplay task procedure directly after experiencing an intrusion, or (b) as the former is not always practical in daily life, they could set aside a time to do the intervention by deliberately recalling the specific memory that is persistent (i.e., gently bring to mind the image that had intruded) and then complete the gameplay task procedure. Monitoring of intrusive memories in the diary is a means by which the participant can notice whether a booster session may be useful.

Participants in the control condition will not be given instructions to repeat the task if they experience an intrusive memory. On balance, we did not consider it justified at this stage in the research to (after a first session) ask healthcare staff during the pandemic to engage in a task hypothesized to be non-active but potentially time consuming.

For participants in the intervention group only, ‘boosters’ (self-administered or researcher-guided) can be initiated by the research team. Researchers will monitor incoming diary data in the intervention condition during Week 1. In the event that a participant continues to report persistent intrusive memories after the initial intervention session, the researcher will contact the participant to:

1) ask them if the intrusion/s was/were from the most recently targeted image (i.e., the image selected in the first guided sessions with the researcher or in a self-administered booster); or a non-targeted image.

If it was a non-targeted image, the researcher will: 2) encourage the participant to use the intervention on their own as soon as possible for this and all remaining non-targeted images (i.e., ‘SMS contact’, see below).

If it was a targeted image, the researcher will: 3) either offer the participant a ‘Full researcher-guided booster’, ‘Phone contact’, or ‘SMS contact’ (see below for more details).

In the current trial, we distinguish between three different types of ‘booster’ approaches:

1) ‘Full researcher-guided booster’ - phone contact between participant and researcher during which the full intervention procedure from Day 1 is repeated, i.e., bringing the target image to mind and at least 20 min of gameplay with mental rotation.
2) ‘Phone contact with researcher’ - phone contact between participant and researcher during which they together explore any difficulties/problem-solve around things that might have made the intervention/booster less effective (e.g., the intrusive image was not clear enough in the mind's eye before the gameplay task; the intrusive memory was not one image but consisted of several discrete visual images and needs to be repeated for each of those; how to engage in mental rotation). After exploring corrective information, the call will end (e.g., after approx. 15 min) and the participant can repeat the intervention procedure self-guided at another time convenient to them.
3) ‘SMS contact with researcher’- a brief SMS from the researcher encouraging the participant to do a self-administered booster dose (without researcher guidance) for any images that still intrude, one image at a time and practical information about how to repeat the intervention (e.g., how to access the game; timings).

#### 2.5.6 Both conditions: completion of daily diary (Week 1 and 5) and follow-ups

Participants in both conditions will be asked to complete an intrusive memory diary and monitor the occurrence of any intrusive memories each day during Week 1 (secondary outcome) and Week 5 post-intervention (primary outcome). Participants will be asked to record the number of intrusive memories they experience each day during the week and to record zero if they did not experience any intrusions. The diary in the electronic platform is accessed via a link received by SMS or email (see 3.1).

Once they have completed the diary for each monitoring period (i.e., on the 7th day of each diary week), participants will be asked to provide overall ratings of intrusion vividness, intrusion-related distress and the extent to which intrusive memories affected their concentration during the past week. They will also be asked how many days, as well as nightshifts, they have worked during the previous week. Additionally, at the end of the Week 5 diary, participants will be asked if they can give an example of (a) how a specific intrusive memory has had a negative impact on their functioning, and (b) if a specific intrusive memory has reduced in number, how this has had a positive impact on their functioning.

Incoming diary data will be monitored by a researcher approximately once a day. Participants in both conditions will be contacted as necessary (e.g., if they cease to complete the digital diary to provide support regarding how to use it).

Participants in both conditions will complete secondary and other outcome measures and note the occurrence of any adverse events on day 7 (1-week). Follow-up measures will also be administered at 1-month, 3-months, and 6-months post-intervention. Data will be collected remotely/digitally (e.g., computer or smartphone) via a secure platform (i.e., SMART-TRIAL [28]). To promote participant retention and data collection, we will be flexible regarding the mode of collection of outcome measures (i.e., we include the option to collect them via telephone, SMS, or on paper if necessary), guided by participant preference.

Approximately a week after completing the primary outcome, participants in the intervention condition will receive a feedback letter thanking them for their participation and including a summary of the number of intrusions they reported at Week 0, Week 1 and Week 5. The letter will also remind participants that they can continue using the intervention if they experience intrusive memories, will include a brief note about the upcoming follow-up assessments, and inform participants that they can contact the research team if they would like to read the publication resulting from the study. Participants in the control condition will receive a similar feedback letter but without their intrusion data or a reminder to continue to use the task. Following an ethics amendment part way through the study, all participants will also be offered a voucher to reimburse them for their time and participation in the weeks after completing the primary outcome. At the end of the study, participants in both groups will also receive a standard letter to again thank them for their participation and provide them with information about publicly available mental health resources, should they wish to seek support.

### 2.6 Training to deliver the intervention and intervention fidelity

At the commencement of the study, research personnel had already been trained to deliver the intervention in-person (i.e., in previous studies) (AG, MK) [22,25]. This training was provided by the originator of the protocol (EH) to MK, and MK then trained AG. MK is a senior investigator with more than three years’ experience in delivering the intervention and training on it.

Adaptations specific to this study required additional peer training within the study team, including for remote and blended digital delivery (rather than in-person delivery), tailored for this study population (health care staff working in the pandemic) [15].

New research personnel (i.e., who join the research team once the study has commenced) will receive extensive training on all aspects of protocol delivery and a focus on guiding participants through the digitalized study procedures. Although most of the instructions and information provided to participants is delivered via prerecorded videos and written instructions in the electronic data collection platform, nonetheless the researcher needs to be able to answer questions and provide individual guidance, both in the first session and in the event of later booster contact.

The content of the training includes the theoretical rationale for the intervention, the components of the intervention, practical aspects of delivering the intervention and control task, how to do boosters, and how to obtain primary outcome data.

The format of training includes informational PowerPoints and an online training course about the intervention developed by the research team, role plays with peers/trainers, real-time/in vivo observations and feedback.

The training will be assessed by competency assessments (conducted by MK) of trainee role plays of intervention delivery, after which MK will provide detailed feedback until the trainee has demonstrated competent delivery of all components of the intervention. Competency in roleplays will be assessed using a rating scale adapted from the Cognitive Therapy Scale – revised [CTS-R; 31], operationalized as a score of at least 4 on each rating of the scale in both intervention and control conditions. MK will also observe each trainee's first c. four cases, and provide reflective feedback.

Throughout the study, supervision will be provided by EH to MK, or by MK to AG/new trainees to maximize adherence to protocol, troubleshoot difficulties, and reflect on how to adapt flexibly to the needs of each specific case. Individual supervision will be given either in real time (e.g., during a participant session) or soon after (on a case-by-case basis), and to prepare for a booster (e.g., looking at the pattern of incoming data together). Ongoing group supervision includes fortnightly peer supervision in a group format led by EH with LS. This includes discussion of cases and learning points with other researchers involved in related studies.

For the components of the procedure delivered by the researcher, fidelity to the protocol will be checked. Specifically, with participants’ consent, phone calls between the researchers and participants can be audio-recorded. An independent researcher (who is not involved in the trial) will rate a random selection (c. 10%) of these recordings for protocol adherence, using a checklist based on a detailed study protocol that specifies each step of the procedure.

## 3 Outcomes

### 3.1 Primary outcome

The primary outcome measure is the number of intrusive memories of traumatic event(s) reported in the diary daily during Week 5 (i.e., for 7 days, from Day 29–35 post-intervention). Participants will receive four links per day (via SMS and email) to record their number of intrusions for morning, afternoon, evening and night respectively, via the electronic platform SMART-TRIAL, starting four weeks after the intervention (i.e., Day 29 post-intervention). Participants will be asked *‘How many intrusive memories did you have during the morning/afternoon/evening/night’* and will be presented with a drop-down menu of 9 possible responses (i.e., ‘0’, ‘1’, ‘2’, ‘3’, ‘4’, ‘5’, ‘6’, ‘7’, ‘more than 7*’).* If a participant selects ‘more than 7’, they can manually enter any number higher than 7.

The link will include a brief description of what intrusive memories are, and the following instructions about how to monitor their occurrence in the diary:

*‘Intrusive memories are IMAGES from a traumatic event that pop suddenly into your mind, when you DO NOT WANT them to. (They are NOT the same as deliberately choosing to think about the event or thinking about it in words.) Please record EVERY intrusive memory you have had - even if it is the same one popping up several times. If you did not have any, please CHOOSE 0.’*

In addition to this brief description, participants will receive more detailed instructions prior to commencing the diary (e.g., in an animated information video about the symptom ‘intrusive memories’, 1:50 min, and via guided researcher support, see also 2.6.1). The diary has been employed in feasibility and pilot work for the current trial in electronic form (i.e., in the SMART-TRIAL platform) [15], as well as in previous work conducted by the group in pen-and-paper form [20]. We note that analysis of the primary outcome will seek to take into account baseline scores, which may be highly variable across participants.

### 3.2 Secondary outcomes

#### 3.2.1 Number of intrusive memories of traumatic event(s) (during Week 0 and Week 1)

Number of intrusive memories of traumatic event(s) reported in a diary daily during Week 0 and Week 1 (as per the primary outcome).

#### 3.2.2 Impact of Event Scale - revised (IES-R), intrusion subscale [32]

Self-report measure that assesses subjective distress after a traumatic event (with reference to study event[s]) in the last week. We will administer the intrusion subscale (8-items). Items are rated on a 5-point scale from 0 (“not at all”) to 4 (“extremely”) and summed; a higher score indicates worse outcome. The IES-R possesses high internal consistency (α = 0.96) as well as high agreement with other measures of posttraumatic stress symptomatology e.g., the PTSD Checklist (r = 0.84) [33]. The IES-R intrusion subscale has been a focus of our previous work [20,25].

#### 3.2.3 Impact of Event Scale - Revised (IES-R), avoidance subscale [32]

Self-report measure that assesses subjective distress after a traumatic event (with reference to study event[s]) in the last week. We will include the avoidance subscale (8-items). Items are rated on a 5-point scale from 0 (“not at all”) to 4 (“extremely”) and summed; a higher score indicates worse outcome.)

#### 3.2.4 Posttraumatic Stress Disorder Checklist 5 (PCL-5), short version [36]

The PCL-5 short version includes 8 items assessing PTSD symptoms in the last month (or in the last week-at 1-week follow-up) [36]. Items are rated on a 5-point scale from 0 (“not at all”) to 4 (“extremely”). Total scores range from 0 to 32; higher scores indicate greater severity. The PCL-5 short version has an extremely high correlation with the original, accounting for 94.1% (r = 0.97) of the variance in the original 20-item validated PCL-5 version [36]. It has been specifically recommended for remote digital assessment after trauma [37].

#### 3.2.5 Self-estimated intrusion frequency to explore convergence with diary measure

##### 3.2.5.1 Intrusion Questionnaire (IQ) - frequency item [47]

A single item measuring the frequency of intrusive/unwanted memories of the traumatic event(s) in the previous week, on a 7-point scale ranging from “never” to “many times a day”. When applicable, participants are asked a follow-up question to specify the exact number of intrusions they have experienced per day.

#### 3.2.6 Characteristics of intrusive memories

##### 3.2.6.1 Intrusion questionnaire (IQ) - characteristics [47]

Five self-rated items measuring characteristics of intrusive/unwanted memories in the previous week; specifically: level of intrusion-related distress, nowness, reliving, disconnectedness and whether different triggers are associated with the intrusive/unwanted memories of the traumatic event(s). Items are rated from 0 to 100; higher scores indicate more distress, nowness, sense of reliving, feelings of disconnection, and more triggers associated with the intrusive/unwanted memories. Previous studies that have used the intrusion questionnaire have included four rating scales: frequency, distress, vividness, nowness. Retest-reliability for these four scales has ranged between r = 0.61 and r = 0.72 [48]. Each item is analysed separately, see Table 1.

##### 3.2.6.2 Distress and vividness of intrusive trauma memories during diary weeks

Two self-rated items assessing the level of distress and vividness associated with intrusions (11-point scales, ranging from 0 to 10) rated within the diary at baseline (Week 0), Week 1 and Week 5; higher scores indicate higher levels of distress and vividness. Each item is analysed separately, see Table 1. This measure is referred to as “Characteristics of intrusive trauma memories” in the Clinical Trials registration.

### 3.3 Other outcomes and assessments

Other outcomes will include general and occupational functioning, as well as the perceived impact of intrusive memories on functioning, items assessing intervention/control task procedures (e.g., to assess adherence to protocol) and feedback items to assess acceptability of the intervention. See Table 1 for an overview of when each measure will be administered.

**Table 1:** Schedule of assessments.

Assessments	Week 0 D-7 to −1	Baseline D1	Task D1	Week 1 D1 to 7	1-week D8	1-month D29	Week 5 D29 to 35	5-weeks D35	3-months D85	6-months D169
***Demographics and participant information***										
Informed consent	X									
Inclusion/exclusion criteria	X									
Randomisation			X							
Demographics		X								
Type and number of traumatic event(s) during the COVID-19 pandemic leading to IMs		X			X	X			X	X
Time the traumatic event(s) leading to IMs occurred		X								
Clinical Background		X								
LEC-5 (prior trauma)		X								
***Primary outcome***										
Number of intrusive memories of traumatic event(s) in diary							X			
***Secondary outcomes***										
Number of intrusive memories of traumatic event(s) in diary	X			X						
IES-R intrusion subscale		X			X	X			X	X
IES-R avoidance subscale		X			X	X			X	X
PCL-5 shortened version		X			X	X			X	X
*Self-estimated intrusion frequency to explore convergence with diary measure*										
IQ-frequency		X			X	X		X	X	X
*Characteristics of intrusive memories*										
IQ characteristics - distress		X			X	X			X	X
IQ characteristics - nowness		X			X	X			X	X
IQ characteristics - reliving		X			X	X			X	X
IQ characteristics - disconnectedness		X			X	X			X	X
IQ characteristics - triggers		X			X	X			X	X
Distress of IMs during diary week		X			X			X		
Vividness of IMs during diary week		X			X			X		
***Other outcomes and assessments***										
*Perceived impact of intrusive memories on functioning*										
Concentration disruption from IMs - level		X			X	X		X	X	X
Concentration disruption from IMs - duration		X			X			X		
Concentration and memory difficulties										X
Impact of IMs on occupational functioning		X			X	X			X	X
Impact of IMS on functioning in other areas		X			X	X			X	X
SCI-02		X			X	X			X	X
Examples of IM impact (open-ended)*								X		
*Perceived functioning and social support*										
Social support after traumatic event		X			X	X			X	X
Self-rated health		X			X	X			X	X
Questions related to work situation		X			X	X			X	X
Sick leave		X			X	X			X	X
SEQ - stress subscale		X			X	X			X	X
Letting go of work-related thoughts		X			X	X			X	X
Moral stress at work		X			X	X			X	X
SWEBO – burnout subscale		X								X
Coping (open-ended)		X								
WHODAS 2.0										X
*Other cognitive assessments*										
Appraisals of IMs – psychological problems		X			X	X			X	X
Appraisals of IMs – negative self-evaluations		X			X	X			X	X
TPQ – past		X			X	X			X	X
TPQ – present		X			X	X			X	X
TPQ - future		X			X	X			X	X
FSQ – vividness									X	
FSQ – positivity									X	
FSQ – perspective									X	
*Assessments related to procedures*										
Adverse Events					X	X			X	X
Credibility/Expectancy questionnaire			X							
Subjective Units of Distress (SUDS)			X							
List of intrusive memories (only intervention arm)*			X							
Adherence to intervention/control task instructions*			X							
Number of booster sessions delivered by researcher*							X			
Number of days/nights at work during intrusion diary weeks*		X			X			X		
*Feedback to assess acceptability and improve materials and procedures for implementation*										
Feedback questionnaire						X				
Open-ended feedback questions*			X		X	X		X	X	X

*Note.* *assessments not listed in CTR as they are assessments related to procedures rather than outcome measures or have open ended responses. Abbreviations: IMs = intrusive memories; LEC-5 = Life Events Checklist for DSM-5, IES-R = Impact of Event Scale – Revised, SCI-02 = Sleep Condition Indicator two-item version, PCL-5 = Posttraumatic Stress Disorder Checklist for DSM-5, SEQ=Stress Energy Questionnaire, SWEBO=Scale of Work Engagement and Burnout, WHODAS 2.0 = World Health Organization Disability Assessment Schedule 2.0, IQ=Intrusion questionnaire, TPQ = Time Perspective Questionnaire, FSQ=Future Self Questionnaire.

#### 3.3.1 Perceived impact of intrusive memories on functioning

##### 3.3.1.1 Self-rated concentration disruption associated with intrusive memories – level and duration

Rating scale derived from Holmes et al. (2017) [3]**.** One item measures the level of concentration disruption associated with the intrusions (‘How much did your intrusive memories disrupt your concentration during the previous week?‘) on an 11-point scale (from 0 to 10); a higher score indicates greater disruption. Another item measures the estimate Self-estimated intrusion frequency to explore convergence with diary measured duration of concentration disruption of each intrusion (‘When you have an intrusive memory, for how long does it disrupt your concentration [in minutes]?‘; <1 min, 1–5 min, 5–10 min, 10–30 min, 30–60 min, > 60 min).

##### 3.3.1.2 Concentration and memory difficulties [38,39]

Eleven items assess the extent of concentration and memory difficulties during the previous four weeks, using a 5-point scale (1 “Virtually every day” to 5 “Never”). Total scores range from 11 to 55; higher scores indicate less concentration and memory difficulties.

##### 3.3.1.3 Self-rated impact of intrusive memories on functioning

Rating scale derived from Iyadurai et al. (2019) [2]***.*** Two items assess the extent to which intrusive memories have an impact on occupational functioning or daily functioning in other areas of life (e.g., social, housework, parenting, etc), using an 11-point scale (0 “no impact” to 10 “extreme impact”) and a free text response field to provide details. Each item is analysed separately, see Table 1. This measure is referred to as “Self-rated functioning” in the Clinical Trials registration.

##### 3.3.1.4 Sleep Condition Indicator (SCI-02) [34]

Two items assess sleep – i.e., participants (i) rate the extent to which they experienced poor sleep (with reference to the study event(s)) on a 5-point scale (from “not at all” to “very much”), and (ii) indicate how many nights they experienced sleep problems per week on a 5-point scale (from 0 to 1 nights to 5–7 nights) as used in our earlier work [35]. Each scale is reverse scored and summed, with a higher total score indicative of better sleep. The test–retest reliability and intraclass correlation coefficient (ICC) have been reported as r = 0.68 and ICC = 0.68 respectively [34]. The SCI-02 is highly correlated with the full eight-item version of the SCI (r = 0.80) [34]. This measure is referred to as “Self-rated sleep ratings” in the Clinical Trials registration.

##### 3.3.1.5 Examples of impact of intrusive memories on functioning

Two bespoke free text response fields which ask participants to provide an example of how a specific intrusive memory has had a negative impact on functioning, and if a specific intrusive memory has reduced, how this has had a positive impact on functioning.

#### 3.3.2 Perceived functioning and social support

##### 3.3.2.1 Social support after traumatic event

One item measuring perceived social support, asking participants to indicate how much social support they received after the traumatic event(s), rated on an 11-point scale (0 “no support” to 10 “a lot of support”).

##### 3.3.2.2 Self-rated health (SRH)

One item assessing perceived health on a 7-point scale (from “very good” to “very bad”) [40]. The scale is reverse scored, high scores indicate good outcomes.

##### 3.3.2.3 Questions related to work situation [41]

Three free response questions (e.g., which type of health care do you work with right now? (only administered at Day 1) and two free response questions on whether the work situation changed and if yes, how.

##### 3.3.2.4 Sick leave [41]

Two bespoke items measuring the total number and the number of full work days on sick leave because of reason for seeking health care. Higher numbers indicate more sick leave. Each item is analysed separately, see Table 1.

##### 3.3.2.5 Stress Energy Questionnaire (SEQ), stress subscale [42]

Three items measuring the number of times participants have felt stressed, pressured, or tense at work during the previous week on a 5-point scale (from “never” to “several times per day”). The three items are summed; higher sum scores indicate higher levels of stress. In a recent study with nurses, the internal consistency reliability was α = 0.96 [43]. Additionally, one item measures difficulties in letting go of work-related thoughts during leisure time (from “very rarely or never” to “very often or always”) [44], and another question asks whether the abovementioned difficulties are due to participants’ intrusive memories (yes/no response).

##### 3.3.2.6 Moral stress at work [41]

Five items assess how much moral stress one feels at work, using a 4-point scale ranging from 1 (“strongly agree”) to 4 (“strongly disagree). All items are summed, with higher scores indicative of lower levels of moral stress.

##### 3.3.2.7 Scale of Work Engagement and Burnout (SWEBO), burnout subscale [45]

Nine items measuring symptoms of burnout on three subscales: exhaustion, disengagement, and inattentiveness. Items are rated on a 4-point scale, from 1 (“not at all”) to 4 (“all the time”) and summed; a higher sum score indicates a higher level of overall burnout. Reliability scores for the burnout dimension range from r = 0.77 to 0.88 [45].

##### 3.3.2.8 Coping

Two bespoke free response questions assessing participants’ perceived coping during the COVID-19 pandemic (i.e., “Based on your experiences and from your perspective, are there any specific situations or factors during the COVID-19 pandemic, that you think made it particularly difficult for you to cope?” and “Based on your experiences and from your perspective, are there any specific factors which you think have made it easier for you to, cope with the COVID-19 pandemic and its consequences?”).

##### 3.3.2.9 WHODAS 2.0

12 self-rated items assessing functioning in six life domains: 1) cognition, 2) mobility, 3) personal care 4) relations, 5) daily activities and 6) participation in society. Each item is rated on a 5-point scale, from 1 (“none”) to 5 (“extreme or cannot do”) and summed; a higher sum score indicates worse functioning. An additional three questions are asked at the end regarding the frequency and impact of these items. WHODAS 2.0 has shown high internal consistency (α = 0.83 to 0.92) and adequate construct validity [46].

#### 3.3.3 Other cognitive assessments

##### 3.3.3.1 Appraisals of intrusive memories [49]

Six self-report items (11-point scale from 0 to 100) measuring appraisals of having intrusions on two subscales: psychological problems (α = 0.94) and negative self-evaluations (α = 0.81) [50]. Items for each subscale are summed, with higher values indicative of worse appraisals [50].

##### 3.3.3.2 Time Perspective Questionnaire (TPQ) [51]

Eight self-report items measuring participants’ time perspective on three subscales: past perspective (α = 0.85 to 0.87), present perspective (α = 0.78 to 0.83), and future perspective (α = 0.71 to 0.75) [51]. All items are rated on a 5-point scale, from 1 to 5, and summed per subscale; higher values indicate a greater sense of past/present/future time perspective.

##### 3.3.3.3 Future Self Questionnaire (FSQ), shortened version, based on [52]

A free response question asking participants to imagine their future self-identity (e.g., ‘I will be a mother”). Participants will also be asked to describe a mental image of this identity, and rate the vividness (from 1 “not vivid at all” to 10 “very vivid”) and positivity (from 1 “very negative” to 10 “very positive”) of the image. Participants will also rate the perspective from which they view the mental image (“through own eyes” or “as if seeing oneself”). Participants will make each of these ratings twice, to indicate their responses both before and after the traumatic event(s). Each item is analysed separately, see Table 1.

#### 3.3.4 Assessments related to procedures

##### 3.3.4.1 Adverse events

A free response question in which participants indicate whether they have experienced any health issues since their last contact with the study team.

##### 3.3.4.2 Credibility/expectancy questionnaire, adapted from Devilly & Borkovec (2000) [53]

Before the intervention or control task, participants will rate the extent to which they consider the intervention/control task credible via five items, on an 11-point scale (from 0 to 10). For example, one item asks participants, “How successful do you think the task will be in preventing/reducing your intrusive memories?”. All items will be summed; a higher score indicates greater credibility. The credibility/expectancy questionnaire possesses high internal consistency (α = 0.84 - 0.85) and good test–retest reliability (r = 0.83) for the whole scale [53].

##### 3.3.4.3 Subjective units of distress (SUDS)

A single item that asks participants to rate their current level of distress on an 11-point scale from 0 (“no distress at all”) to 10 (“worst imaginable distress”), to be completed three times during the intervention/control procedure.

##### 3.3.4.4 List of intrusive memories (only intervention arm)

As part of the intervention, participants will list a brief description of the content of their intrusive memories in six free response fields. More than six responses can be entered if necessary.

##### 3.3.4.5 Adherence to intervention/control task instructions

Adherence to the intervention/control task instructions will be assessed via three multiple choice items asking participants the most important thing they did before completing the task, what they mainly focused on during the task, and the approximate amount of time they spent completing it. An additional single item asks participants to rate the extent to which they agree with the statement ‘During the past 20 min, I followed the instructions’ on an 11-point scale (from 0 “not at all” to 10 “extremely”). For the intervention group, two additional items ask participants to rate the vividness of their trauma memory before playing Tetris, and whether they turned off “ghost piece” during game play.

##### 3.3.4.6 Number of booster sessions delivered by researcher

The number of booster sessions delivered by a researcher will be recorded in the platform, along with the date of each booster session.

##### 3.3.4.7 Number of days/nights at work during week of intrusion monitoring

Two separate items asking participants how many days/nights they had worked during the previous week.

#### 3.3.5 Feedback to assess acceptability and improve materials and procedures for implementation

##### 3.3.5.1 Feedback questionnaire about participation

At 1-month follow-up, participants will be asked to provide four ratings about the task they completed (intervention or control): i.e., how simple it was to complete, how upsetting it was, how acceptable they found it, and whether they would recommend it to a colleague or friend who has had a similar experience, all on a scale of 0 (not at all) to 10 (very much). Participants will also be asked whether they have done the task on their own (yes/no; if they say yes they are asked to indicate how many times), and whether they have received any treatment as a result of the traumatic event since they commenced participation in the study (yes/no). If participants indicate they have received any treatment, they will be asked to describe it. Participants will also be asked if they have told others (e.g., friends, colleagues) about the task (yes/no), and also asked if they have any other comments.

##### 3.3.5.2 Open ended feedback questions

After completing their randomly assigned task on Day 1, participants will complete five free response questions about the feasibility of the task, the instructions, and their experience of doing the task. Additionally, at each follow-up time point, participants will be asked to provide any comments they have about the study procedure, or indicate how the study could be improved in the future.

### 3.4 Adverse events

We will screen for adverse events (at 1-week, 1-month, 3-month, and 6-month follow-ups) using a brief self-report form (‘Have you had any health problems since the last contact’ [yes/no], if yes is answered: ‘Briefly describe which:‘) used by our local clinical trials unit (KTA).

Any adverse events reported (whether spontaneously reported by the participant to study personnel or during follow-ups in the electronic platform SMART-TRIAL) will be registered in an adverse event log. Adverse events will be classified as serious or not. Adverse events will be assessed for severity (mild, moderate or severe) and relationship to intervention (not related, unlikely, possible, probable, definite) by the Principal Investigator. Any actions taken will be logged.

### 3.5 Monitoring of additional treatments

Participants will be asked at baseline whether they have ever been diagnosed with and have ever received treatment for any mental health problem/s (including depression, anxiety or PTSD).

At one-month follow-up participants will be asked (on the Feedback Questionnaire about participation) whether they have received any treatment since they did the task for the first time (e.g., psychological treatment or medication) because of the traumatic event/s. If participants indicate they have received treatment, they will be asked to provide details in an open-ended response.

## 4 Statistical considerations

### 4.1 Data analysis

This is a between-subjects design in which we are interested in between-group comparisons post-intervention. The primary outcome (number of intrusive memories) is based on count data, which is not typically normally distributed [54]. We expect large variability in the number of intrusive memories reported across participants, which may also vary as the pandemic progresses, and we seek to take into account baseline rates.

Main analyses will be based on intention-to-treat; i.e., will utilize available data from all randomized participants. A statistical analysis plan (SAP) will be preregistered (e.g., on the Open Science Framework, OSF) prior to data analysis. The statistician conducting the analyses will be blind to condition (e.g., groups will be labelled only as ‘A’ and ‘B’ in the analysis data file). Analyses will be conducted in appropriate statistical software and we aim to make data analysis code publicly available (e.g., on the OSF).

Corrections for multiple testing are not required for the primary outcome, which includes only one comparison at a single time-point. We also plan to explore data such as participants’ qualitative responses regarding their experience in the study and impressions using the intervention/control task. Numerical outliers will be removed in cases where data was not a legitimate part of the data generation process (e.g., errors in data entry, invalid or implausible responses). Missing data will be addressed using multiple imputation. Sensitivity analyses will be conducted to assess the potential impact of missing data on the results (recommended by the National Research Council Panel on Handling Missing Data in Clinical Trials [56]).

### 4.2 Sample size estimation

We anticipate recruiting a total of 164 participants (*n* = 82 per condition) in order to detect a medium effect, based on findings for the number of intrusive memories at Week 5 in our pilot study, mean intervention = 0.28 (SD = 0.57) versus mean control = 2.89 (SD = 6.43) [25], and factoring in potential attrition. Based on this between group difference (*d* = 0.57, equivalent of ∼0.5 standard deviation units), power of 90% and alpha of 0.05, we would require 65 participants per group (130 completers in total, see also our earlier planned study [26]). The attrition rate of 12.2% in the pilot trial [25] would suggest aiming to recruit 146 participants in the current trial. However, given the challenges of the current COVID-19 pandemic and their potential to compromise retention, we have opted to take a more conservative approach and estimate an attrition rate of 20%; accordingly, our estimated enrolment would be 164 participants. If, for example attrition is lower than expected we may well enroll fewer than the estimated 164 participants. Decisions will be made with statistical and data monitoring input and other individuals/groups overseeing the trial.

### 4.3 Data management

Included participants will be pseudonymized (coded) by assigning a unique numeric identifier. Participant data will be treated confidentially and the system used for data collection and study handling is secure and password-protected. Self-report data will be entered by participants directly into an electronic Case Report Form (eCRF) form (encrypted secure and GDPR compliant electronic data collection platform); other data (e.g. eligibility criteria, number of booster sessions) is entered into the eCRF by the researchers. Any digital documents that include identifying information (e.g., the electronic signed Informed Consent Forms/recordings) will be stored separately from participant data on a secure password protected university server; any paper documents will be stored in a locked fireproof safe. The principle investigator will have continued access to the final dataset. Participants provide their informed consent that their pseudonymized data may be made available for secondary research (e.g., via the OSF). We aim to share pseudonymized data according to the FAIR Guiding Principles (i.e., such that data is findable, accessible, interoperable, and reusable) [58]. More details regarding data management will be specified in a data management plan created using the ‘DMPonline’ system.

## 5 Ethical considerations

The study will be performed in accordance with the study protocol and the ethical principles of the World Medical Association (WMA) Declaration of Helsinki (as amended by the 64th WMA General Assembly, Fortaleza, Brazil, October 2013) [59], and aims to follow Good Clinical Practice (GCP) guidelines.

The study has received ethical approval from the Swedish Ethical Review Authority (approval no. 2020–03085). Modifications to the protocol (e.g., to include an audio-recorded verbal informed consent process; adapt inclusion criteria; adapt advertising materials; add additional measures) have been approved (amendments no. 2020–06399, 2020–06600, 2021–01651, 2021–03691) and we plan to request approval as necessary for any modifications in the future.

The study was preregistered in the Clinical Trials Registry (clinicaltrials.gov) on 07-07-2020 (no. NCT04460014). This protocol aims to follow the Standard Protocol Items: Recommendations for Interventional Trials (SPIRIT) guidelines [60]; see checklist in Appendix 1. The trial sponsor is Karolinska Institutet, Sweden (contact information: emily.holmes@ki.se). The study includes a trial steering committee chaired by Professor Peter McEvoy (independent member). The advisory committee at project start includes Dr. Ann Rudman, Dr. Katarina Göransson, and Dr. Anna Hörberg (representatives of our participant group). An independent clinical trials unit (Karolinska Trial Alliance, KTA) will monitor the study (e.g. ethical considerations, study protocol, primary outcome data) to promote compliance with Good Clinical Practice (GCP). Staff on the study will receive training in GCP.

Participants will provide their informed consent remotely (as attested by scanned paper consent form or audio-recorded verbal consent) prior to commencing the study. Prior to giving consent, participants will receive information about the nature of the study, its purpose, expected duration, the benefits, and risks involved. Participants will be informed that their participation is voluntary and that they can withdraw their consent at any time without providing a reason. Participants will receive this information in written form (i.e. via email or downloaded from the study webpage) and will be given as much time as they need to read study information materials and consider study participation. Prior to taking informed consent, the investigator or a member of the research team will answer any questions about this information or any aspect of study participation via phone/video call. In this phone/video call eligibility for study participation will be assessed as well.

Our ethical procedures for remote recruitment during the pandemic have been developed in collaboration with representatives of our participant group (including practicing nurses, ambulance nurses, nurse researchers) and were piloted prior to commencing the RCT [15].

We plan to communicate trial results as publications in peer-reviewed scientific journals, through presentations at national and international scientific conferences and internal meetings at the hospital/university. We plan to follow internationally recognised authorship guidelines (e.g. guidelines of the International Committee of Medical Journal Editors, ICMJE).

## 6 Discussion

Recurrent intrusive memories are a common psychological consequence of exposure to traumatic events. Not only are such memories distressing in their own right, they can also have a significant and negative impact on functioning. Accordingly, intrusive memories represent an important clinical target, and interventions which effectively reduce the number of intrusive memories following traumatic events are needed. Interventions that are brief, can be delivered remotely and within the initial days or weeks after trauma, and that once learnt can be used in a self-guided manner, have the potential to protect psychological well-being and prevent disrupted functioning in both the short and longer-term.

The need for such interventions is nowhere more apparent than in healthcare staff – a group that has been (and continues to be) exposed to significant rates of traumatic events in the course of their work during repeated waves of the COVID-19 pandemic [61,62] and their aftermaths. Exploratory pilot work with our novel cognitive task intervention targeting intrusive memories with patients who presented to the ED following trauma has provided an initial indication that participants who received the intervention reported fewer intrusive memories compared to control 5 weeks post-intervention [25]. Further, another pilot/co-design study with three nurses working during the COVID-19 pandemic showed that a digitalized and remotely-delivered (guided) form of the intervention was well received by this target population (healthcare staff) [15].

As the next step in this line of investigation, we have here outlined the protocol for an RCT: the first investigation of the effects of a blended digital and remotely-delivered form of this brief cognitive task intervention on the number of intrusive memories after traumatic event(s) in healthcare staff working during the COVID-19 pandemic. The intervention will be delivered using a blend of various digital tools with guided (remote) support from a researcher with the aim of fitting study procedures around the reality of staff's working lives under the pandemic. This format confers multiple practical advantages: it is brief, can be completed anywhere, and is repeatable independently by staff during ongoing/further trauma exposure. Further, from the outset, all aspects of the conduct and implementation of the trial have been developed in close collaboration with experts by lived experience – that is, healthcare staff working at the frontline during the pandemic. Another advantage of the intervention for this population is that it does not require discussion of the trauma in detail, and thus participants do not need to become significantly distressed. Moreover, it is notable that across all of the studies evaluating this intervention conducted to date, no adverse events related to study procedures have been reported.

Raising an important final consideration, we emphasize the unprecedented context in which this trial is being carried out. Since the conception and commencement of the trial, the severity and impact of the COVID-19 pandemic in Sweden escalated to an extent that could not have been foreseen. At the time of submitting this manuscript (May 2021), Sweden was at crisis point – having just recorded the highest number of daily COVID-19 cases in Europe, and with hospital resources significantly overstretched [63]. It is plausible that the increasing severity of this situation may result in an increase in the number of intrusions, and the degree of intrusion-related distress, reported by participants enrolled in the trial as it progresses over time. At the time of submitting the revised version of this manuscript, and after the vaccination program in Sweden rolled out, there had been a drop in the number of COVID-19 cases.

Such significant and varying circumstances may need to be taken into account in interpreting the data, to enable meaningful conclusions to be drawn. Further, should the trial yield a null finding, we would be hesitant to simply conclude that the cognitive task intervention per se is ineffective. Rather, a null finding may indicate a lack of evidence for *this* particular blended version of the brief cognitive task intervention (comprised of digital components and guided, remote support), delivered to *this* particular population of Swedish healthcare staff, during an ongoing pandemic which escalated in intensity whilst the trial was underway. Similarly, evidence of fewer intrusive memories would not necessarily indicate that the positive effects of this version of the intervention would generalize to other populations, without further adaptations to specific contexts. Nonetheless, should this form of the intervention result in fewer intrusive memories in this population, the outcomes will pave the way for implementation of a simple, novel and flexible approach that is highly scalable and ready for dissemination – to reduce long-term psychological distress in healthcare staff in the current pandemic and well beyond.

## Trial status

Recruitment commenced on September 30, 2020; the trial is ongoing.

## Funding sources

This project was mainly supported by grants to EAH from the 10.13039/501100004359Swedish Research Council (2017–00957 and 2020–00873), the 10.13039/100001275Oak Foundation (OCAY-18-442), 10.13039/501100002706AFA Insurance (200342), and the 10.13039/100010431Lupina Foundation. LS was supported by a 10.13039/100000001Swiss National Science Foundation grant (P2BEP1_184378) and a Thunberg Fellowship by the Swedish Collegium for Advanced Study. MK was supported by a FO Medical Psychology grant from 10.13039/501100004047Karolinska Institutet and Karolinska University Hospital. BG was supported by a grant to EAH from the 10.13039/100001275Oak Foundation (OCAY-18-442). KG was supported by a grant from FO Akut, Karolinska University Hospital. AR was supported by a grant from 10.13039/501100002706AFA Insurance (200311). Funders were not involved in the study design, collection, analysis and interpretation of data; writing of the report; and/or decision to submit the report for publication.

## Author contributions

LS: Conceptualization, Resources, Writing – original draft, Writing – review & editing, Project administration.

MK: Conceptualization, Resources, Writing – review & editing, Project administration.

BG: Methodology, Writing – review & editing.

AG: Resources, Writing – review & editing.

KG: Writing – review & editing, Funding acquisition.

AR: Writing – review & editing, Funding acquisition.

OD: Writing – review & editing.

VL: Writing – review & editing.

AH: Writing – review & editing.

AF: Writing – review & editing.

EAH: Conceptualization, Methodology, Writing – review & editing, Supervision, Project administration, Funding acquisition.

MM: Conceptualization, Writing – original draft, Writing – review & editing.

## Declaration of competing interest

E.A.H. reports serving on the board of trustees of the charity MQ: Transforming Mental Health but receives no remuneration for this role. E.A.H. receives royalties from books and occasional fees for workshops and invited addresses; she receives occasional consultancy fees from the Swedish agency for health technology assessment and assessment of social services.

The other authors have no competing interest to declare.

## References

[1] Holmes E.A., O'Connor R.C., Perry V.H., Tracey I., Wessely S., Arseneault L., Ballard C., Christensen H., Cohen Silver R., Everall I., Ford T., John A., Kabir T., King K., Madan I., Michie S., Przybylski A.K., Shafran R., Sweeney A., Worthman C.M., Yardley L., Cowan K., Cope C., Hotopf M., Bullmore E. (2020). Multidisciplinary research priorities for the COVID-19 pandemic: a call for action for mental health science, the lancet. Psychiatry.

[2] Iyadurai L., Visser R.M., Lau-Zhu A., Porcheret K., Horsch A., Holmes E.A., James E.L. (2019). Intrusive memories of trauma: a target for research bridging cognitive science and its clinical application. Clin. Psychol. Rev..

[3] Holmes E.A., Ghaderi A., Eriksson E., Lauri K.O., Kukacka O.M., Mamish M., James E.L., Visser R.M. (2017). 'I can't concentrate': a feasibility study with young refugees in Sweden on developing science-driven interventions for intrusive memories related to trauma. Behav. Cognit. Psychother..

[4] American Psychiatric Association (2013).

[5] Kleim B., Bingisser M.B., Westphal M., Bingisser R. (2015). Frozen moments: flashback memories of critical incidents in emergency personnel. Brain and behavior.

[6] Kang L., Ma S., Chen M., Yang J., Wang Y., Li R., Yao L., Bai H., Cai Z., Xiang Yang B., Hu S., Zhang K., Wang G., Ma C., Liu Z. (2020). Impact on mental health and perceptions of psychological care among medical and nursing staff in Wuhan during the 2019 novel coronavirus disease outbreak: a cross-sectional study. Brain Behav. Immun..

[7] Lai J., Ma S., Wang Y., Cai Z., Hu J., Wei N., Wu J., Du H., Chen T., Li R., Tan H., Kang L., Yao L., Huang M., Wang H., Wang G., Liu Z., Hu S. (2020). Factors associated with mental health outcomes among health care workers exposed to Coronavirus Disease 2019. JAMA Netw. Open.

[8] Tan B.Y.Q., Chew N.W.S., Lee G.K.H., Jing M., Goh Y., Yeo L.L.L., Zhang K., Chin H.K., Ahmad A., Khan F.A., Shanmugam G.N., Chan B.P.L., Sunny S., Chandra B., Ong J.J.Y., Paliwal P.R., Wong L.Y.H., Sagayanathan R., Chen J.T., Ng A.Y.Y., Teoh H.L., Ho C.S., Ho R.C., Sharma V.K. (2020). Psychological impact of the COVID-19 pandemic on health care workers in Singapore. Ann. Intern. Med..

[9] Greenberg N., Weston D., Hall C., Caulfield T., Williamson V., Fong K. (2021). Mental health of staff working in intensive care during COVID-19. Occup. Med. (Lond.).

[10] Laposa J.M., Alden L.E. (2003). Posttraumatic stress disorder in the emergency room: exploration of a cognitive model. Behav. Res. Ther..

[11] Laposa J.M., Alden L.E., Fullerton L.M. (2003). Work stress and posttraumatic stress disorder in ED nurses/personnel. J. Emerg. Nurs..

[12] Mealer M., Burnham E.L., Goode C.J., Rothbaum B., Moss M. (2009). The prevalence and impact of post traumatic stress disorder and burnout syndrome in nurses. Depress. Anxiety.

[13] Wild J., El-Salahi S., Degli Esposti M. (2020). The effectiveness of interventions aimed at improving well-being and resilience to stress in first responders: a systematic review. Eur. Psychol..

[14] Singh L., Espinosa L., Ji J.L., Moulds M.L., Holmes E.A. (2020). Developing thinking around mental health science: the example of intrusive, emotional mental imagery after psychological trauma. Cognit. Neuropsychiatry.

[15] Singh L., Kanstrup M., Depa K., Falk A.C., Lindstrom V., Dahl O., Goransson K.E., Rudman A., Holmes E.A. (2021). Digitalising a brief intervention to reduce intrusive memories of psychological trauma for healthcare staff working during COVID-19: an exploratory pilot study with nurses. JMIR Form Res.

[16] Holmes E.A., James E.L., Coode-Bate T., Deeprose C. (2009). Can playing the computer game "Tetris" reduce the build-up of flashbacks for trauma? A proposal from cognitive science. PLoS One.

[17] James E.L., Bonsall M.B., Hoppitt L., Tunbridge E.M., Geddes J.R., Milton A.L., Holmes E.A. (2015). Computer game play reduces intrusive memories of experimental trauma via reconsolidation-update mechanisms. Psychol. Sci..

[18] Baddeley A.D., Andrade J. (2000). Working memory and the vividness of imagery. J. Exp. Psychol. Gen..

[19] Horsch A., Vial Y., Favrod C., Harari M.M., Blackwell S.E., Watson P., Iyadurai L., Bonsall M.B., Holmes E.A. (2017). Reducing intrusive traumatic memories after emergency caesarean section: a proof-of-principle randomized controlled study. Behav. Res. Ther..

[20] Iyadurai L., Blackwell S.E., Meiser-Stedman R., Watson P.C., Bonsall M.B., Geddes J.R., Nobre A.C., Holmes E.A. (2018). Preventing intrusive memories after trauma via a brief intervention involving Tetris computer game play in the emergency department: a proof-of-concept randomized controlled trial. Mol. Psychiatr..

[21] Kessler H., Holmes E.A., Blackwell S.E., Schmidt A.C., Schweer J.M., Bücker A., Herpertz S., Axmacher N., Kehyayan A. (2018). Reducing intrusive memories of trauma using a visuospatial interference intervention with inpatients with posttraumatic stress disorder (PTSD). J. Consult. Clin. Psychol..

[22] Kanstrup M., Kontio E., Geranmayeh A., Olofsdotter Lauri K., Moulds M.L., Holmes E.A. (2020).

[23] Iyadurai L., Hales S.A., Blackwell S.E., Young K., Holmes E.A. (2020). Targeting intrusive imagery using a competing task technique: a case study. Behav. Cognit. Psychother..

[24] Kanstrup M., Rudman A., Göransson K., Andersson E., Lauri K.O., Rapoport E., Sunnergård L., Bragesjö M., Andersson E., Iyadurai L., Holmes E.A. (2021). Reaching people soon after a traumatic event: an exploratory observational feasibility study of recruitment in the emergency department to deliver a brief behavioral intervention via smartphone to prevent intrusive memories of trauma. Pilot Feasibility Stud.

[25] Kanstrup M., Singh L., Göransson K.E., Widoff J., Taylor R.S., Gamble B., Iyadurai L., Moulds M.L., Holmes E.A. (2021). Reducing intrusive memories after trauma via a brief cognitive task intervention in the hospital emergency department: an exploratory pilot randomised controlled trial. Transl. Psychiatry.

[26] Kanstrup M., Singh L., Goransson K.E., Gamble B., Taylor R.S., Iyadurai L., Moulds M.L., Holmes E.A. (2021). A simple cognitive task intervention to prevent intrusive memories after trauma in patients in the Emergency Department: a randomized controlled trial terminated due to COVID-19. BMC Res. Notes.

[27] Gamble B., Depa K., Holmes E.A., Kanstrup M. (2021). Digitalizing a brief intervention to reduce intrusive memories of psychological trauma: qualitative interview study. JMIR Ment Health.

[28] Smart-Trial. https://www.smart-trial.com.

[29] Agren T., Hoppe J.M., Singh L., Holmes E.A., Rosén J. (2021). The neural basis of tetris gameplay: implicating the role of visuospatial processing. Curr. Psychol.: A Journal for Diverse Perspectives on Diverse Psychological Issues.

[30] Radio S., Rummet Filosofiska (2018). https://sverigesradio.se/avsnitt/1073596#:%7E:text=Hur%20har%20den%20analytiska%20filosofin,och%20sociolog%20Carl%2DG%C3%B6ran%20Heidegren.%202021.

[31] Blackburn I., James I., Milne D., Baker C., Standart S., Garland A., Reichelt F. (2001). The revised cognitive therapy scale (CTS-R): psychometric properties. Behav. Cognit. Psychother..

[32] Weiss D.S., Marmar C.R., Wilson J.P., Keane T.M. (1997). Assessing Psychological Trauma and PTSD: A Handbook for Practitioners.

[33] Creamer M., Bell R., Failla S. (2003). Psychometric properties of the impact of event scale - revised. Behav. Res. Ther..

[34] Luik A.I., Machado P.F., Siriwardena N., Espie C.A. (2019). Screening for insomnia in primary care: using a two-item version of the Sleep Condition Indicator. Br. J. Gen. Pract..

[35] Luik A.I., Iyadurai L., Gebhardt I., Holmes E.A. (2019). Sleep disturbance and intrusive memories after presenting to the emergency department following a traumatic motor vehicle accident: an exploratory analysis. Eur. J. Psychotraumatol..

[36] Price M., Szafranski D.D., van Stolk-Cooke K., Gros D.F. (2016). Investigation of abbreviated 4 and 8 item versions of the PTSD Checklist 5. Psychiatr. Res..

[37] Price M., van Stolk-Cooke K., Brier Z.M.F., Legrand A.C. (2018). mHealth solutions for early interventions after trauma: improvements and considerations for assessment and intervention throughout the acute post-trauma period. mHealth.

[38] Rudman A., Arborelius L., Dahlgren A., Finnes A., Gustavsson P. (2020). Consequences of early career nurse burnout: a prospective long-term follow-up on cognitive functions, depressive symptoms, and insomnia. EClinicalMedicine.

[39] Nilsson L.-G., BÄCkman L., Erngrund K., Nyberg L., Adolfsson R., Bucht G., Karlsson S., Widing M., Winblad B. (1997). The betula prospective cohort study: memory, health, and aging. Aging Neuropsychol. Cognit..

[40] Fylkesnes K., Førde O. (1982). Determinants and dimensions involved in self-evaluation of health. Soc. Sci. Med..

[41] Rudman A., Omne-Pontén M., Wallin L., Gustavsson P.J. (2010). Monitoring the newly qualified nurses in Sweden: the longitudinal analysis of nursing education (LANE) study. Hum. Resour. Health.

[42] Hadzibajramovic E., Ahlborg G., Grimby-Ekman A., Lundgren-Nilsson Å. (2015). Internal construct validity of the stress-energy questionnaire in a working population, a cohort study. BMC Publ. Health.

[43] Frögéli E., Rudman A., Gustavsson P. (2019). The relationship between task mastery, role clarity, social acceptance, and stress: an intensive longitudinal study with a sample of newly registered nurses. Int. J. Nurs. Stud..

[44] Kecklund G., Åkerstedt T. (1992). The pattern of slow wave activity in spontaneously occurring long sleep. J. Sleep Res..

[45] Hultell D., Gustavsson J.P. (2010). A psychometric evaluation of the scale of work engagement and burnout (SWEBO). Work.

[46] Axelsson E., Lindsäter E., Ljótsson B., Andersson E., Hedman-Lagerlöf E. (2017). The 12-item self-report World health organization disability assessment schedule (WHODAS) 2.0 administered via the internet to individuals with anxiety and stress disorders: a psychometric investigation based on data from two clinical trials. JMIR Ment Health.

[47] Hackmann A., Ehlers A., Speckens A., Clark D.M. (2004). Characteristics and content of intrusive memories in PTSD and their changes with treatment. J. Trauma Stress.

[48] Speckens A.E., Ehlers A., Hackmann A., Clark D.M. (2006). Changes in intrusive memories associated with imaginal reliving in posttraumatic stress disorder. J. Anxiety Disord..

[49] Newby J.M., Moulds M.L. (2011). Do negative appraisals and avoidance of intrusive memories predict depression at six months?. Int. J. Cognit. Ther..

[50] Newby J.M., Moulds M.L. (2010). Negative intrusive memories in depression: the role of maladaptive appraisals and safety behaviours. J. Affect. Disord..

[51] Holman E.A., Silver R.C., Mogle J.A., Scott S.B. (2016). Adversity, time, and well-being: a longitudinal analysis of time perspective in adulthood. Psychol. Aging.

[52] Rathbone C.J., Conway M.A., Moulin C.J. (2011). Remembering and imagining: the role of the self. Conscious. Cognit..

[53] Devilly G.J., Borkovec T.D. (2000). Psychometric properties of the credibility/expectancy questionnaire. J. Behav. Ther. Exp. Psychiatr..

[54] Coxe S., West S.G., Aiken L.S. (2009). The analysis of count data: a gentle introduction to Poisson regression and its alternatives. J. Pers. Assess..

[56] National Research Council (2010).

[58] Wilkinson M.D., Dumontier M., Aalbersberg I.J., Appleton G., Axton M., Baak A., Blomberg N., Boiten J.-W., da Silva Santos L.B., Bourne P.E., Bouwman J., Brookes A.J., Clark T., Crosas M., Dillo I., Dumon O., Edmunds S., Evelo C.T., Finkers R., Gonzalez-Beltran A., Gray A.J.G., Groth P., Goble C., Grethe J.S., Heringa J., ’t Hoen P.A.C., Hooft R., Kuhn T., Kok R., Kok J., Lusher S.J., Martone M.E., Mons A., Packer A.L., Persson B., Rocca-Serra P., Roos M., van Schaik R., Sansone S.-A., Schultes E., Sengstag T., Slater T., Strawn G., Swertz M.A., Thompson M., van der Lei J., van Mulligen E., Velterop J., Waagmeester A., Wittenburg P., Wolstencroft K., Zhao J., Mons B. (2016). The FAIR Guiding Principles for scientific data management and stewardship. Sci. Data.

[59] World Medical Association (2013). https://www.wma.net/policies-post/wma-declaration-of-helsinki-ethical-principles-for-medical-research-involving-human-subjects/.

[60] Chan A.-W., Tetzlaff J.M., Gøtzsche P.C., Altman D.G., Mann H., Berlin J.A., Dickersin K., Hróbjartsson A., Schulz K.F., Parulekar W.R., Krleža-Jerić K., Laupacis A., Moher D. (2013). SPIRIT 2013 explanation and elaboration: guidance for protocols of clinical trials, BMJ. Br. Med. J..

[61] L. Bergman, A.-C. Falk, A. Wolf, I.-M. Larsson, Registered nurses' experiences of working in the intensive care unit during the COVID-19 pandemic, Nurs. Crit. Care n/a(n/a) doi: 10.1111/nicc.12649. PMC8242789 33973304

[62] Daniels J., Ingram J., Pease A., Wainwright E., Beckett K., Iyadurai L., Harris S., Donnelly O., Roberts T., Carlton E. (2021). The COVID-19 clinician cohort (CoCCo) study: empirically grounded Recommendations for forward-facing psychological care of frontline doctors. Int. J. Environ. Res. Publ. Health.

[63] Henley J. (2021).

