# Supplementary material for: A first remotely-delivered guided brief intervention to reduce intrusive memories of psychological trauma for healthcare staff working during the ongoing COVID-19 pandemic: Study protocol for a randomised controlled trial
Source: Contemp Clin Trials Commun. 2022 Jan 12;26:100884. doi: 10.1016/j.conctc.2022.100884 (PMC8752164; doi:10.1016/j.conctc.2022.100884)
Supplement: Multimedia component 1 [file mmc1.pdf]

Information till forskningspersonerna (v. 3.3 2021-08-30)

Studiens titel: *”Enkel Kognitiv Uppgift efter Trauma under COVID-19 - sjukvårdspersonal ’EKUT-P’ – en randomiserad kontrollerad studie”*

Vi vill fråga dig om du vill delta i ett forskningsprojekt.

Här får du information om projektet och om vad det innebär att delta.

Vad är en psykologiskt traumatisk händelse under COVID-19 pandemin?
Efter en traumatisk händelse är det vanligt att sjukvårdspersonal som arbetat t.ex. på sjukhus, inom äldreomsorg, eller ambulansverksamhet upplever *återkommande, ofrivilliga och påträngande minnesbilder* av det som hände.

Exempel på sådana situationer kan vara:
- Ett traumatiskt eller tragiskt dödsfall för en patient.
- Situationer där vården av en patient misslyckades.
- Situationer där vården av en patient inte fungerade som planerat.

Kan enkla kognitiva uppgifter hjälpa mot påträngande minnen?
Vår forskargrupp försöker hitta interventioner som kan minska förekomsten av påträngande minnen. Vi har sett lovande initiala resultat till exempel för patienter i akutsjukvård. Medicinsk sjukvårdspersonal har själva efterfrågat om de kan delta i vår forskning. Vi vill därför nu se om enkla kognitiva uppgifter kan hjälpa också för dig som är medicinsk sjukvårdspersonal.

Syftet med den här studien är att undersöka om en enkel kognitiv uppgift kan förebygga och minska påträngande minnesbilder. Vi hoppas att studien kan bidra till utvecklingen av nya behandlingsmetoder för personer som varit med om traumatiska händelser, till exempel de som varit med om traumatiska händelser under COVID-19.

Du är välkommen att kontakta oss för att ställa frågor om det är något du undrar över! Våra kontaktuppgifter finns nedan.

Vem kan delta i studien?
Vi söker deltagare som är *sjukvårdspersonal.*

Du kan delta i studien om du:

*Är 18 år eller äldre

*Har haft klinisk tjänstgöring under COVID- 19 pandemin på vårdinrättning på sjukhuset (t.ex IVA, intermediärvård, vårdavdelning, ambulans). *(Detta omfattar personal som arbetat för sjukhuset under denna tidsperiod och har haft patientkontakt)*

*Har varit med om minst en traumatisk händelse i samband med ditt kliniska arbete som sjukvårdspersonal under COVID-19.
(Exempel på sådana händelser kan vara ett traumatiskt eller tragiskt dödsfall för en patient; hot eller våld mot sjukvårdspersonal; situationer där vården av en patient misslyckades eller inte fungerade som planerat; händelser som innebar förhöjd risk för COVID-19 smitta; bevittna händelser som omgärdar en kollega som blivit sjuk eller avlidit av COVID-19.)

*Denna händelse/dessa händelser på arbetet innebar faktiskt eller risk för död, allvarlig skada eller sexuellt våld för dig eller för någon annan (till exempel traumatiskt eller tragiskt dödsfall för en patient)

*Den traumatiska händelsen/händelserna har skett under tiden sedan COVID-19 pandemin startade

*Minns den traumatiska händelsen/händelserna

*Upplever besvärande påträngande minnen (av en traumatisk händelse i samband med ditt arbete som klinisk sjukvårdspersonal under COVID-19)

*Har upplevt minst två sådana påträngande minnen av en arbetsrelaterad traumatisk händelse under veckan innan tillfället för inklusion/exklusion

*Kan och vill kort benämna eller kortfattat skriva ner dessa påträngande minnen (utan att gå in på dem i någon detalj)

*Kan läsa och prata svenska flytande

*Är alert och orienterad

*Har tillgång till en internetansluten smartphone

*Klarar av rent fysiskt att använda din smartphone

*Vill och kan ge ditt informerade samtycke till deltagande och slutföra studieproceduren

*Vill och kan genomföra uppföljningarna och kontaktas under det att studien pågår

Du kan inte delta i studien om du hade:

*Medvetandeförlust > 5 minuter i samband med den traumatiska händelsen

*Pågående intoxikation under den traumatiska händelsen eller i samband med att du anmäler ditt deltagande

Hur går studien till?
Studien är helt digitaliserad och sköts via din dator eller smartphone. Kontakt med forskargruppen sker via telefon, email eller videosamtal.

Om du deltar i studien kommer du att slumpas till att göra en av två enkla kognitiva uppgifter på din smartphone. Du kan genomföra uppgiften självständigt men har möjlighet att få hjälp från oss.

För att vi ska kunna utvärdera resultaten och veta om uppgiften har hjälpt dig kommer du att få föra en enkel digital ’dagbok’ över antalet påträngande minnen du har av den traumatiska händelsen/händelserna. Dagboken fyller du i veckan innan samt under den första och den femte veckan efter det att du gjort uppgiften första gången.

Du kommer också att få fylla i olika frågeformulär vid uppföljningar efter 1 vecka och vid 1, 3, och 6 månader.

Vi kommer att hålla kontakt med dig under ditt deltagande i studien. Du kan få påminnelser om att fylla i dagboken och formulären. Vi försöker att anpassa oss till det sätt som passar dig bäst för att hålla kontakt med forskargruppen.

Vi kan komma att vilja göra en ljudinspelning av forskaren vid något av de tillfällen som ni har kontakt. Detta är för att försäkra oss om att forskaren levererar studien på ett korrekt sätt till dig som deltar. Du kan välja att avstå ifrån ljudinspelning om du vill.

Forskningshuvudman för projektet är Karolinska Institutet. Med forskningshuvudman menas den organisation som är ansvarig för studien.

Deltagandet är frivilligt
Det är frivilligt att delta. Om du väljer att delta får du fylla i ett digitalt samtycke till deltagande, och kan behålla den skriftliga informationen.

Du kan när som helst avbryta din medverkan utan att behöva ange någon orsak. Du kan kontakta oss via uppgifterna nedan. Det är frivilligt att besvara alla frågor du får under studiens gång.

Finns det några för- eller nackdelar med att delta i studien?
Eventuella fördelar kan vara att få något att sysselsätta sig med som distraktion eller avkoppling, och att det kan vara intressant att delta i en forskningsstudie och bidra till ökad kunskap om hur vi kan hjälpa personer att må bättre efter traumatiska händelser.

Eventuella nackdelar kan vara att det upplevs som jobbigt att besvara frågor om det som har hänt och ditt psykiska mående, men det är frivilligt att besvara alla frågor du får under studiens gång, och du kommer inte att behöva gå in på några detaljer om den traumatiska händelsen. Uppgifterna som ingår i studien kan *inte* liknas vid psykologiska terapier där man pratar om traumatiska händelser i detalj.

Vad händer med mina uppgifter?
För att kunna genomföra forskningen samlar vi in och registrerar information som du lämnar till oss. Dina personuppgifter och övriga uppgifter om dig som samlas in skyddas av bestämmelser om sekretess enligt offentlighets- och sekretesslagen. Dina svar och resultat kommer att behandlas så att inte obehöriga kan ta del av dem. Om information du gett bedöms som att du eller någon annan löper risk för påtaglig skada, så kan kontakt initieras med relevant professionell vårdinstans för att hjälpa dig. Dina personuppgifter kommer att sparas och lagras på ett säkert sätt, till exempel krypterat i en säker plattform. Ansvarig forskare är Professor Emily Holmes.

Den fortsatta vetenskapliga bearbetningen och rapporteringen av informationen du gett oss muntligen och i frågeformulär kommer att ske utan att enskilda personer kan identifieras.
Vi kanske väljer att citera något som du har sagt ordagrant i en publikation som kommer från denna studie. Om vi gör så, kommer identifierande information vara borttagen.

Den information vi fått från dig införs i ett forskningsregister. Karolinska Institutet är ansvarig forskningshuvudman och personuppgiftsansvarig.
Dina personuppgifter behandlas på Karolinska Institutet (KI), och lagras i 10 år efter projektavslut. Kontaktperson är ansvarig forskare för genomförandet av projektet, Emily Holmes, emily.holmes@ki.se / 079-0680431 alt. 079-0680432 (studietelefon).

Data kan komma att läggas ut i öppna vetenskapliga databaser (t.ex. Open science framework) enligt rådande vetenskaplig praxis. Om data läggs ut i öppna vetenskapliga databaser kommer den att vara kodad för att undvika att enskilda personer kan identifieras. Det innebär att din forskningsdata kan tillgängliggöras för andra som är intresserade av denna forskning efter att studien är avslutad, men bara efter att information som kan identifiera dig eller andra är borttagen. Forskning är resurskrävande och finansieras ofta med statliga medel och av ideella organisationer i syfte att gagna befolkningen på bästa möjliga sätt. Därför uppmuntrar allt fler aktörer att data görs öppet tillgänglig i vetenskapliga öppna databaser för att maximera nyttan av forskningen. Detta eftersom det tillåter oss att besvara

forskningsfrågor utöver de frågor som de insamlats för samt att det möjliggör utvärdering av forskningens kvalitet.

KI är en statlig myndighet och har en skyldighet att bland annat följa reglerna för allmänna handlingar, myndigheters arkiv och offentlig statistik. KI kommer därför även att behandla personuppgifterna på de sätt som krävs för att kunna följa gällande lagstiftning. Du har rätt att begära tillgång till och, när så är möjligt, få rättelse eller radering av personuppgifter eller begränsning av den behandling som rör dig eller möjlighet att invända mot behandlingen. Om du har synpunkter på KIs behandling av dina uppgifter kan du kontakta dataskyddsombudet, dataskyddsombud@ki.se. Om du är missnöjd med det svar du får har du rätt att ge in klagomål till Datainspektionen, som är tillsynsmyndighet, datainspektionen@datainspektionen.se eller 08-657 61 00.

Du kommer att kunna ta del av resultat från hela studien i form av en vetenskaplig publikation, men inte se dina egna resultat. Om du vill ta del av resultaten från studien, kontakta forskargruppen.

Försäkring och ersättning
Försäkring för ditt deltagande i studien gäller via Karolinska Institutet. I anslutning till uppföljningen efter 5 veckor erhåller du som tack för ditt deltagande dittills i studien ett presentkort på H&M, 300 kr.

Vem har granskat studien?
Den här studien har fått etiskt godkännande av Etikprövningsmyndigheten: DNR 2020-03085

Forskningshuvudman: Karolinska Institutet.
Ansvarig forskare: Professor Emily Holmes
Epost: emily.holmes@ki.se
Studietelefon: 079-0680431 alt. 079-0680432.
Postadress: Institutionen för klinisk neurovetenskap, Karolinska Institutet, 171 77 Stockholm

Såhär kommer du i kontakt med forskargruppen: *ekut@cns.ki.se*

Stort tack för din tid, varmt välkommen att höra av dig med frågor.

Samtycke till deltagande (v. 3.3 2021-08-30)

INFORMERAT SAMTYCKE TILL DELTAGANDE I STUDIEN KAN GES SKRIFTLIGEN VIA DENNA BLANKETT ELLER VIA MUNTLIGT INFORMERAT SAMTYCKE I TELEFONSAMTAL MED FORSKAREN, SOM SPELAS IN FÖR DOKUMENTATION

Studiens titel: *”Enkel Kognitiv Uppgift efter Trauma under COVID-19 – sjukvårdspersonal ’EKUT-P’ – en randomiserad kontrollerad studie”*
Ansvarig forskare: Professor Emily Holmes
Dnr för studiens etikgodkännande: 2020-03085

Jag har fått information om studien, och har haft möjlighet att ställa frågor och få dem besvarade. Jag får behålla den skriftliga informationen. ☐ JA

Jag förstår att mitt deltagande är frivilligt och att jag kan avbryta mitt deltagande när som helst utan att behöva ange skäl. ☐ JA

Jag förstår att mina uppgifter behandlas konfidentiellt, med undantag för att kontakt kan initieras med relevant professionell vårdinstans i händelse av att information jag ger bedöms som att jag eller någon annan löper risk för påtaglig skada. ☐ JA

Jag förstår att min data kan göras tillgänglig för andra i öppna vetenskapliga databaser (t.ex Open Science Framework), enligt rådande vetenskaplig praxis och efter att information som kan identifiera mig eller andra är borttagen. ☐ JA

Jag förstår att citat från mig kan komma att användas i publikationer från forskargruppen, efter det att information som kan identifiera mig eller andra är borttagen. ☐ JA

Jag ger mitt samtycke för att delta i studien: *”Enkel Kognitiv Uppgift efter Trauma under COVID-19 – sjukvårdspersonal ’EKUT-P’ – en randomiserad kontrollerad studie”* ☐ **JA**

Jag samtycker till att uppgifter om mig behandlas på det sätt som beskrivs i forskningspersonsinformationen. ☐ **JA**

Separat samtycke gäller för nedan alternativ (valfria):
Jag samtycker till ljudinspelning av delar av mitt studiedeltagande i studien ovan:
☐ **JA** ☐ **NEJ**

Jag kan tänka mig att bli kontaktad igen av forskargruppen för att bli tillfrågad om att eventuellt delta i andra studier i framtiden:
☐ **JA** ☐ **NEJ**

Deltagares namn:____

______________________________ ____
Ort och datum **Signatur**

Forskare som inhämtat samtycke:____

______________________________ ____
Ort och datum **Signatur**
